# Aorto-esophageal fistula caused by fishbone ingestion: a case report on staged endovascular and endoscopic treatment

**DOI:** 10.1186/s12876-021-01624-9

**Published:** 2021-02-02

**Authors:** Yu-Yan Zhang, Shan Li, Xiang-Lei Yuan, Bing Hu

**Affiliations:** Department of Gastroenterology, West China Hospital, Sichuan University, Sichuan Province, No. 37 Guoxue Alley, Wuhou District, Chengdu, 610041 China

**Keywords:** Case report, Esophageal fistula, Foreign bodies, Endoscopy, Endovascular procedures

## Abstract

**Background:**

Aorto-esophageal fistula (AEF) caused by foreign bodies ingestion is a rare but devastating disorder. Thoracic endovascular aortic repair (TEVAR) has become a widely accepted intervention for treating aorto-esophageal fistulas. As for post-TEVAR esophageal defect, secondary esophagectomy has been the recommended choice for most of the AEFs, but there is no general consensus with regard to the need of secondary surgeries for patients in the absence of clear signs of reinfection or bleeding. We herein presented a case of an AEF caused by fishbone ingestion, after successful TEVAR, the esophageal lesion was closed endoscopically.

**Case presentation:**

A 38-year-old male presented with esophageal fistula for 4 months. He was diagnosed with AEF because of Chiari's triad after fishbone ingestion 4 months ago. Emergency thoracic aortic stent implantation was done, and given broad spectrum antibiotics and blood transfusion. His symptoms were improved, and discharged with an esophageal fistula left to heal itself. Nevertheless, after 4 months, re-examination of esophago-gastro-duodenoscopy revealed that the diameter of the fistula was changed from 3 to 6 mm. He was then admitted to our hospital for esophageal fistula repair. Laboratory examinations and chest computed tomography showed no signs of active infection, and endoscopic closure of the fistula was achieved with 4 clips. After that, he was discharged and gradually returned to normal diet.

**Conclusion:**

For AEFs in the absence of active infection with repaired aorta but persistent esophageal fistula, endoscopic closure by endoclips might be an effective treatment choice.

## Background

Aorto-esophageal fistula (AEF) caused by foreign bodies (FB) ingestion is a rare but devastating disorder. On account of initial exsanguination and secondary mediastinal infection and subsequent sepsis, the mortality rate of aorto-esophageal fistula formed by FB ingestion is higher than 90% [1, 2]. The current first-line treatment strategy is to control fatal bleeding by TEVAR in the urgent phase, followed by radically debridement of the contaminated area as well as the aortic/esophageal reconstruction in the semi-urgent phase [3, 4]. However, here is no general consensus with regard to the need for secondary surgeries following the successful use of TEVAR in the absence of clear signs of reinfection or bleeding [5]. We herein presented our successful experience in managing an AEF patient, by describing a combined endovascular and endoscopic approach.

## Case presentation

### Chief complaints

A 38-year-old male who underwent TEVAR for AEF was admitted to our hospital with esophageal fistula lasting from 4 months.

### History of present illness

**Fig. 1:**
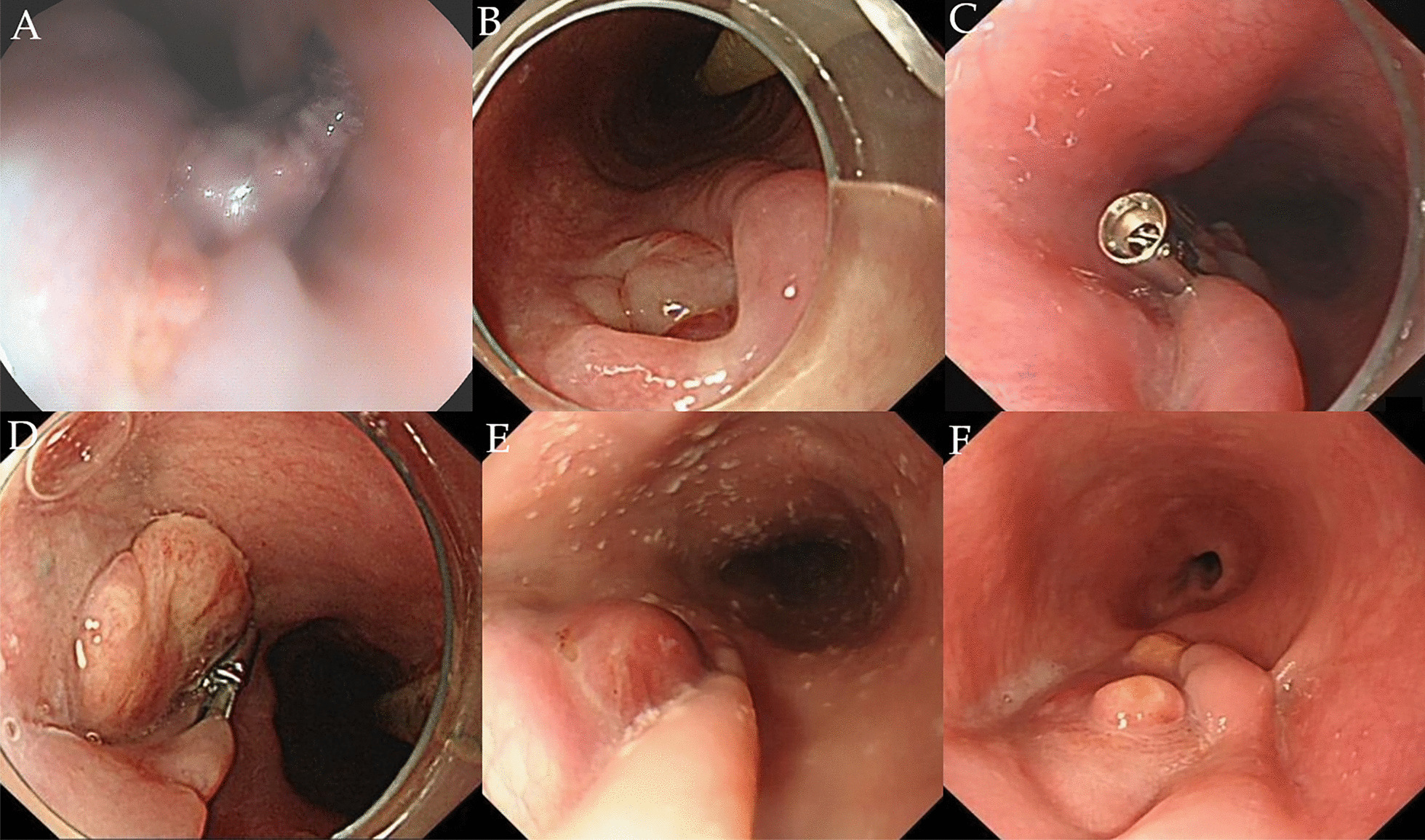
Endoscopy view: **a** an esophageal fistula of 30 cm from the incisors, about 3 mm in diameter with influx of bloody fluids, and accompanied with a hematoma and hyperemic mucosa; **b** after 4 months, the esophageal fistula was about 6 mm in diameter; **c** endoclips were used to close the fistula; **d** the fistula was closed at 1-month follow-up; **e** endoscopy revealed completely healed fistula and fungal esophagitis at 3-months follow-up; **f** at 1-year follow-up, the fistula was healed with no signs of infection

**Fig. 2:**
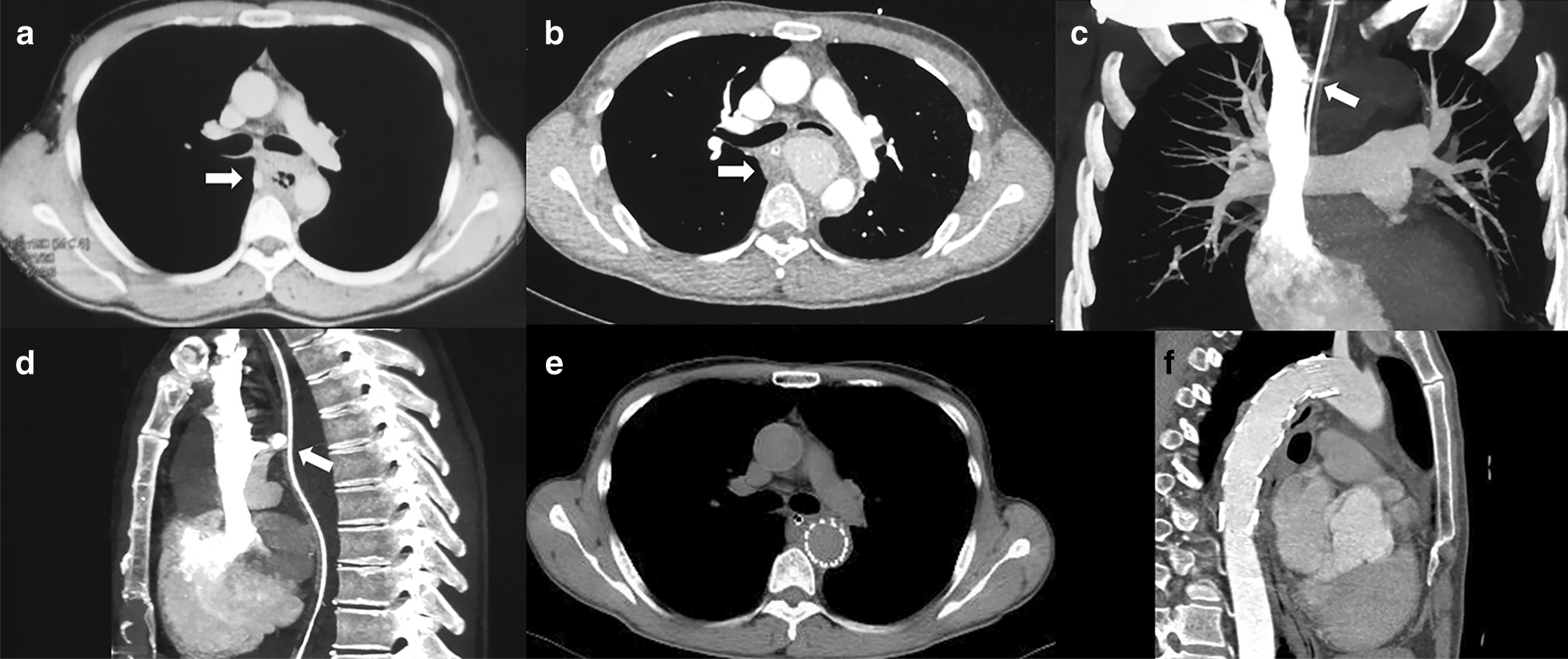
Computed tomography: **a** air bubbles in the mediastinum (white arrow); **b** mediastinal abscess in the mediastinum (white arrow); **c** coronal CT angiography showed contrast leakage from aorta toward esophagus (white arrow); **d** sagittal CT angiography demonstrated a pseudoaneurysm with a primary entry tear at the thoracic aorta (white arrow); **e** except for the radiopaque of aortic stent and nasojejunal tube, no signs of infection were found; **f** sagittal CT angiography did not find any abnormality, except for the radiopaque of aortic stent

The patient while eating was stuck by a fishbone, and he consciously swallowed it by drinking water 4 months ago. On day 6, he developed chest pain, on day 8 melena, on day 9 fever, and then he was admitted to hospital. Esophagogastroduodenoscopy (EGD) showed an esophageal fistula of 30 cm from the incisors, about 3 mm in diameter with influx of bloody fluid, and accompanied by a hematoma and hyperemic mucosa (Fig. 1a). Chest CT revealed air bubbles in the mediastinum, suggesting esophageal perforation (Fig. 2a). Neither EGD nor CT found any signs of fish bones. Broad spectrum antibiotics and nasojejunal feeding was given. The patient after that developed sudden massive hematemesis and melena in 7 days. Enhanced CT showed formation of mediastinal abscess, pseudoaneurysm and contrast material extravasation from the aorta to esophagus (Fig. 2b–d), confirming the diagnosis of AEF. A thoracic endovascular stent graft (Zenith TX2 TAA ref ZTEG-2PT-32-160; Cook, Denmark) in 32-mm proximal and 28-mm distal diameter and 160-mm length was then implanted. His symptoms were improved, and discharged with a nasojejunal tube, and the esophageal fistula was assumed to heal on its own. About 4 months later, EGD again displayed the esophageal fistula of about 0.6 cm in diameter (Fig. 1b). He was then admitted to our hospital for esophageal repair. In the past 4 months, he ate through nasojejunal tube and lost 6 kg weight, and had no fever, hematemesis or melena.

### History of past illness

The patient had no previous medical history and no specific family history.

### Physical examination

At admission, the patient’s temperature was 36.5 °C, heart rate was 96 bpm, respiratory rate was 20 breaths per minute, blood pressure was 125/80 mmHg, height was 178 cm, and weight was 57.5 kg. No other special findings were reported.

### Laboratory examinations

Routine blood tests, including white blood cell count of 7.56 × 10^9^/L, mainly neutrophils 68.7%, hemoglobin concentration 134 g/L were normal. The blood biochemistries were also normal. The procalcitonin concentration of 0.13 ng/mL was shown to be slightly increased, and fecal occult blood was weakly positive.

### Imaging examinations

Except for the radiopaque of well-positioned aortic stent and nasojejunal tube, there were no signs of infection on chest plain CT (Fig. 2e).

### Final diagnosis

Aorto-esophageal fistula (after thoracic aortic stent implantation).

### Treatment

The patient had no signs of active infection and the aortic stent functioning was well, but the fistula was growing. Hence, 4 endoclips were used to close the fistula (Fig. 1c).

### Outcome and follow-up

**Fig. 3:**
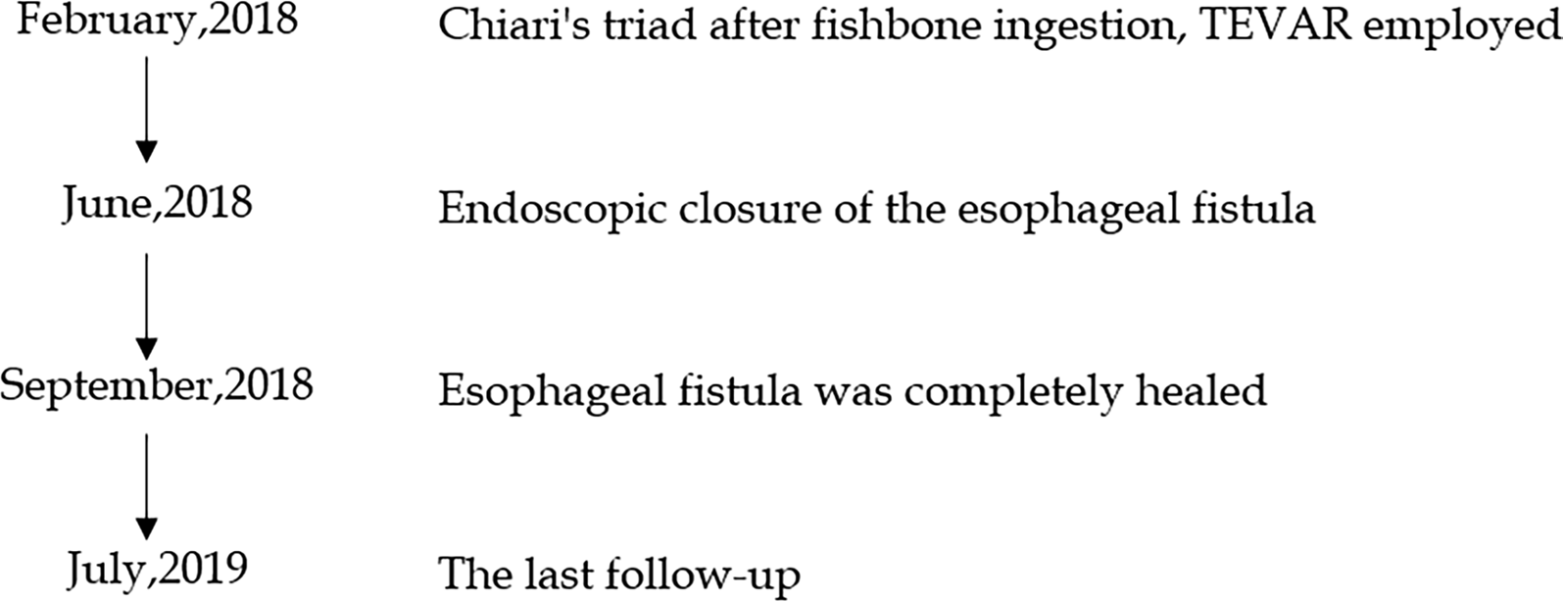
Timeline of the patient with aorto-esophageal fistula

No postoperative complications occurred. He was discharged from the hospital the next day. At 1-month follow-up, EGD revealed the closure of the fistula (Fig. 1d). At 3-months follow-up, EGD revealed completely healed fistula and suspicious fungal esophagitis (Fig. 1e). Brush biopsy did not find any fungus, and so no treatment was given. The nasojejunal tube was removed and the patient was returned to normal diet. At 12 months, the patient had fever, and blood culture detected streptococcus anginosus. Endoscopic brush biopsy found yeast-like fungus, and routine urine test found yeast cells and routine stool test found fungus. Chest CT and angiography revealed no evidence of mediastinitis, aortic stent-graft infection, or new episodes of gastrointestinal bleeding (Fig. 2f). After combined treatment of aztreonam, vancomycin and voriconazole, the patient showed improvement and was discharged from the hospital. Re-examination by EGD revealed no signs of infection (Fig. 1f). In the last year, the patient gained 7 kg weight. The timeline of this case was shown in Fig. 3.

## Discussion and conclusions

In association with life-threatening hemorrhage and infection, AEF is a rare but devastating disorder. Most of the patients present with Chiari's triad, which includes midthoracic pain or dysphagia, a sentinel episode of hematemesis, followed by fatal exsanguination. With the aim to save life, the therapeutic principles of AEF included controlling fatal bleeding, restoring the integrity of aorta and esophagus, treating and preventing infection. In recent years, TEVAR has become a widely accepted intervention for AEFs owing to its definite operative effect in bleeding control and minimal injury in restoring the aortic continuity. However, the use of TEVAR alone for AEF left esophageal perforation untreated, and therefore the patients are at risk of AEF recurrence and/or stent-graft contamination, which is associated with high mid-term mortality [5]. Therefore, TEVAR should be applied as a first life-saving choice for AEF patients, but subsequent treatment is indispensable [3, 4].

In special patients, the esophageal defects were healed by themselves. Tao et al. have reported that a 5 mm esophageal fistula caused by fishbone ingestion is healed on its own after endovascular repair, but the whole process took 1 year and 3 months, another case reported by Shen JY et al. with two esophageal fistulas of 0.2 cm and 0.3 cm in diameter took 6 months to heal by itself [6, 7]. In most of the cases, considering the high risk of infection, most of the authors are in favor of secondary esophagectomy followed by esophageal reconstruction [1, 3, 8]. Due to non-negligible injuries, expenses, and complications associated with open surgery, the pros and cons of esophagectomy should be carefully weighed, especially in patients with the repaired aorta and without any infection or bleeding.

With the development of endoscopy, endoscopic therapy has taken part in the management of AEFs. Luca Mezzetto et al. have reported a case of an AEF who was successfully treated by endoscopic clipping of the esophageal defect before endovascular repair [9]. Esophageal stents have been used for managing AEFs, but the experiences with its use in AEFs are limited as well. A 2017 study summarized the previous application of an esophageal stent in AEF, with 10 cases involved [10]. Compared to esophagectomy, esophageal stent therapy had a more unfavorable prognosis, and so not recommended as a routine for patients with AEF [3]. Meanwhile, esophageal stent placement carries the risks of severe adverse events, including stent migration that requires repositioning or replacement [4]. More importantly, the main purpose of esophageal stent placement in AEF patients involves the prevention of aortic graft contamination as a bridging therapy in the urgent phase [10–12]. In our case, his infection and bleeding were controlled, and his main demand was to return to a normal oral diet. At the same time, as we know, endoclip is an effective choice to close the chronic esophageal perforation and can be used even for persistent fistulas [13]. Therefore, rather than esophageal stent, the endoclips therapy was chosen, as it is a one-shot operation, more convenient, targeted, and cost-efficient.

With no evidence of mediastinitis and aortic stent-graft infection, we inferred that the patient’s fungal esophagitis, and subsequent streptococcal septicemia and systemic fungal infection might be associated with the previous use of broad-spectrum antibiotics, decreased immunity, and long-term nasojejunal feeding. The patient improved after adequate antibiotic treatment. But he was followed up closely. As recommended by the recent reviews, TEVAR should not be considered as a definitive surgery, and similarly, the secondary surgery of graft replacement and esophagectomy should be planned for complete cure, regardless of the level of surgical invasion [3, 4]. Once the symptoms of infection occur in any patient with a history of AEF, especially those without secondary surgery, they should be highly suspected for the possibility of mediastinitis or stent-graft infection and evaluated for secondary surgeries in the clinical setting. Also, lifelong imaging surveillance is mandatory [7].

The combined strategy of endovascular and endoscopic treatment worked well in our case. Certainly, it can only be considered for selected patients. Also, it should be validated with more persuasive studies like case–control studies, whereas it is difficult to achieve by the nature of the rarity of AEF.

TEVAR has gained recognition in treating AEF due to its critical technique to control exsanguination and restore aorta integrity. As for the management of esophageal lesions after TEVAR, endoscopic therapy might be a minimally invasive alternative to surgery in selected patients with small defects and without active infection. Certainly, careful and comprehensive evaluation before the operation and strict intensive follow-up are required.

## Data Availability

All data generated or analyzed during this study are included in this published article.

## Abbreviations

AEF: Aorto-esophageal fistula
FB: Foreign bodies
TEVAR: Thoracic endovascular aortic repair
AEFs: Aorto-esophageal fistulas
EGD: Esophago-gastro-duodenoscopy
CT: Computed tomography
